# First person – Meri Uusi-Mäkelä and Sanna-Kaisa Emilia Harjula

**DOI:** 10.1242/dmm.052321

**Published:** 2025-03-24

## Abstract

First Person is a series of interviews with the first authors of a selection of papers published in Disease Models & Mechanisms, helping researchers promote themselves alongside their papers. Meri Uusi-Mäkelä and Sanna-Kaisa Emilia Harjula are joint first authors on ‘ The inflammasome adaptor *pycard* is essential for immunity against *Mycobacterium marinum* infection in adult zebrafish’, published in DMM. Meri conducted the research described in this article while a PhD student in Mika Rämet's lab at Tampere University, Tampere, Finland. They are now a postdoc in the lab of Birgitta Henriques-Normark at Karolinska Institutet, Stockholm, Sweden, investigating bacterial infection models. Sanna-Kaisa is a postdoc in Mika Rämet's lab at Tampere University, investigating bacterial infection models in zebrafish.


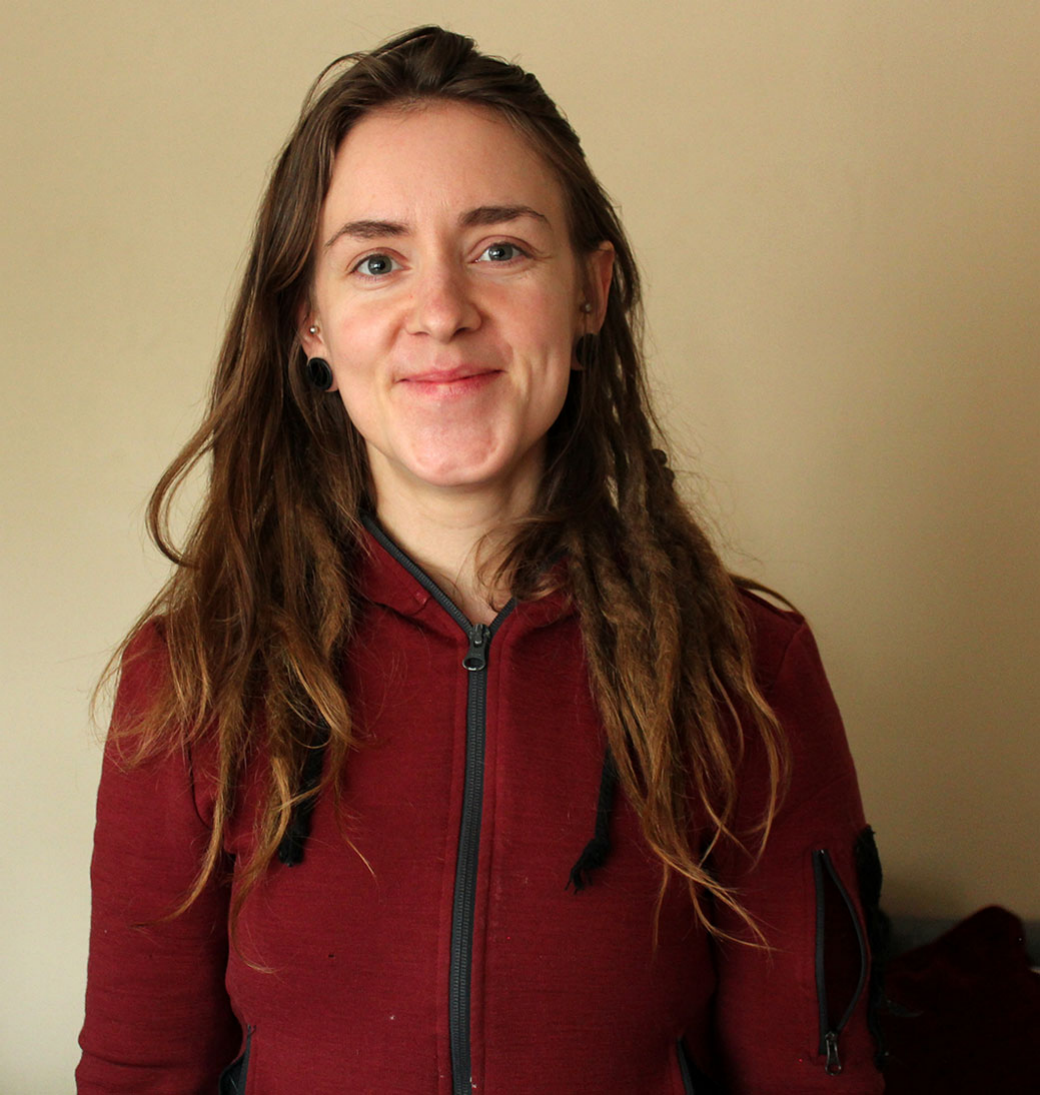


**Meri Uusi-Mäkelä**

**Who or what inspired you to become a scientist?**

While constructing the interview, we found that we were both wanting initially to become school teachers. We agreed that the driving force behind the transformation from a teacher to a researcher was our very stubborn nature and unwillingness to accept the limitations of what we know to be a restrictive boundary rather than something that can gradually be pushed forward. We both adore digging around for new information. During the manuscript preparation process, we found each other just delving into the deep bog of speculation and connecting remote findings to establish connections – something we laugh about a lot nowadays but find still very characteristic of our collaborations.

**What is the main question or challenge in disease biology you are addressing in this paper? How did you go about investigating your question or challenge?**

Our paper aims at finding what is the role of inflammasome signalling in mycobacterial infection. Previous evidence offers very few experiments done on rodent models, which then fail to determine the extent of the immunocompromised state of the subject. Our findings soon pointed to a less established connection between inflammasome and neutrophils – the cell type that is notoriously difficult to study *in vivo*. We found that we can at least offer a transcriptomic scope of how neutrophils are affected by the lack of inflammasome, which seems to be a whole new field to explore.

[…] we were able to describe […] the cellular processes that are compromised in these fish and ultimately point to what could be causing them being sensitive to mycobacterial infections.

**How would you explain the main findings of your paper to non-scientific family and friends?**

We set out to study a gene we know is essential for our defence against bacteria. Using a set of fish models that we ourselves generated, we explored what kind of role this gene plays in defence against tuberculosis in a fish model. We were able to connect the lack of this gene into changes in a cell type called neutrophils, which is essential in the initial steps of infection. Using a more detailed analysis of neutrophils, we were able to describe – to a limited extent – the cellular processes that are compromised in these fish and ultimately point to what could be causing them being sensitive to mycobacterial infections.

**What are the potential implications of these results for disease biology and the possible impact on patients?**

Tuberculosis, while being mostly absent in western world, is a serious problem in large parts of the world. Our results are mostly basic research, so more applied research needs to be done before we can move on to treating patients that suffer from tuberculosis. Despite this, inflammasome would be a very interesting target for drug development, whether we should downplay or stimulate it. For neutrophil research, we have shown that this difficult-to-study cell type can suffer from the lack of inflammasome, which seems to compromise a number of cellular processes upon infection.


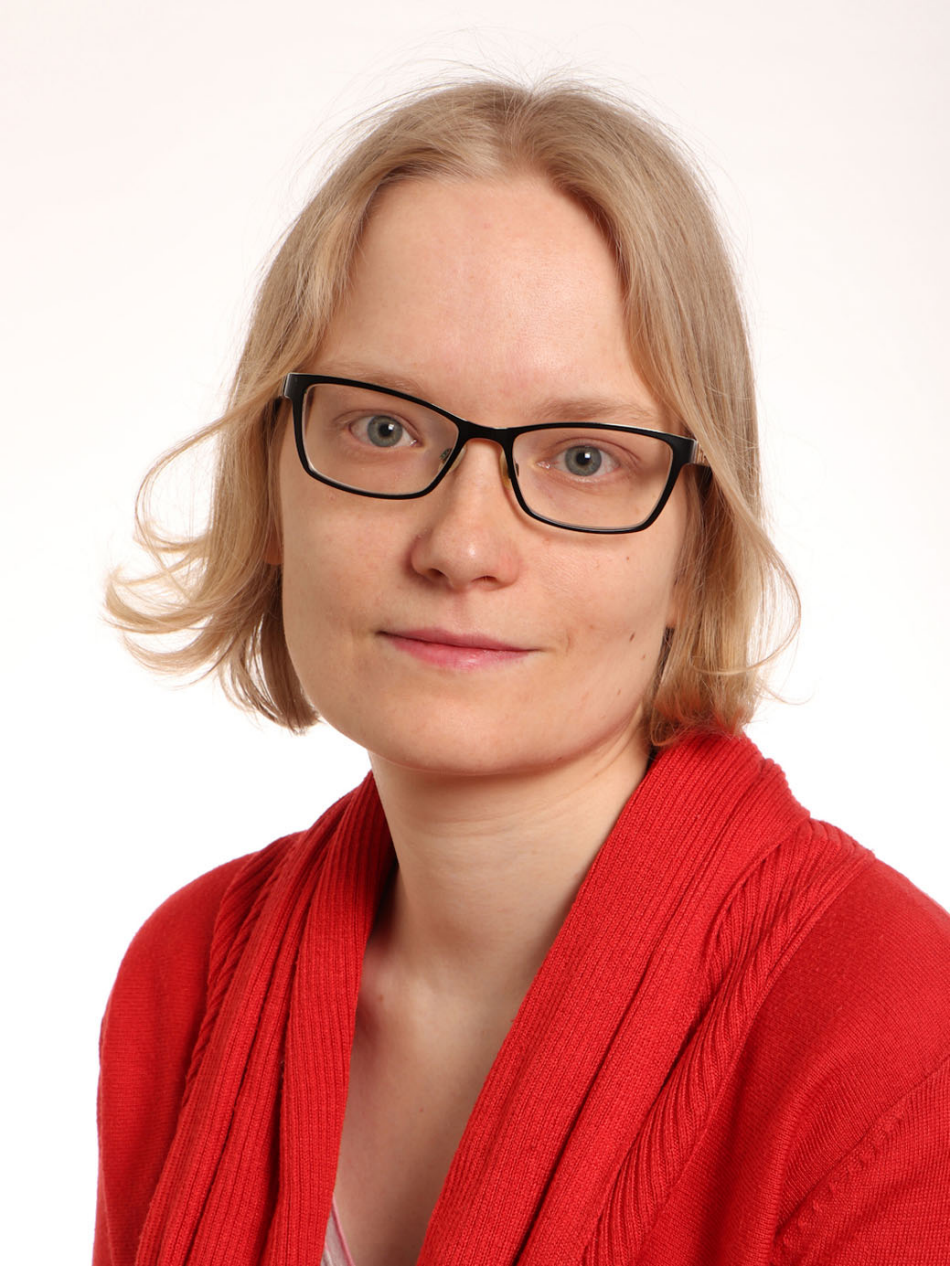


**Sanna-Kaisa Emilia Harjula**

**Figure:**
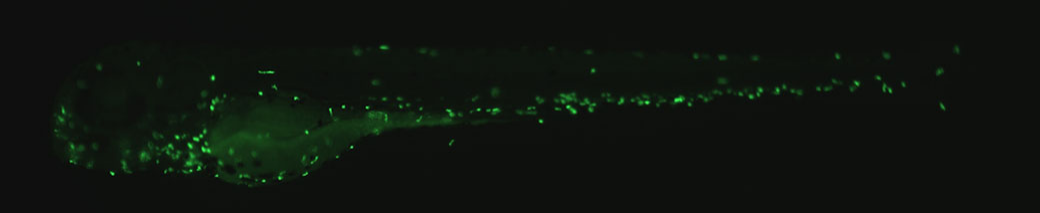
**The brightest fish of the article: *pycard^tpu4/tpu4^ × Tg(mpx:GFP)i114 (AB)* larvae have fluorescent neutrophils**. These larvae were infected with *Mycobacterium marinum* and imaged 1 and 2 days post infection.

**Why did you choose DMM for your paper?**

Disease Models and Mechanisms is the perfect journal for our research, as we are indeed setting out to discover new mechanisms behind tuberculosis with our fish model. As the adult zebrafish model is less explored for modelling human disease, our research highlights some important features of the adult fish, which could be used in studying human infectious diseases.

In an ideal world, less competition would allow us to concentrate on science rather than writing grant applications for weeks and months each year.

**Given your current role, what challenges do you face and what changes could improve the professional lives of other scientists in this role?**

Postdoctoral researchers currently have to compete for their funding. Basic research is still a major driving force behind many advances of medical technology, but finding funding for science that does not necessarily produce new treatments is scarce. This is an important factor that drives postdocs to switch fields and move to industries, leading to our knowledge becoming obsolete. In an ideal world, less competition would allow us to concentrate on science rather than writing grant applications for weeks and months each year. Another case is female researchers who have children, who are forced out of science because they have to simply work more to accommodate childcare and research work.

**What's next for you?**

We are both applying for funding to start our own research groups, or to become junior PIs. We both hope that we can inspire more funders of the splendid ideas that are pouring out of our collaborations.

**Tell us something interesting about yourself that wouldn't be on your CV**

We both love reading and usually discuss how our books are progressing over coffee breaks. Currently there are a lot of kids' books at both our bedsides, and we have found we can discuss these almost as much as the adult versions.

**Is there anything better in science than sitting in a dark room with a fluorescence microscope?**

We have agreed that, if there is, it has yet to be discovered. A pair of headphones or a friend to make notes is perfect company. We also sing a lot of songs, such as the famous ‘I Will Survive’ or ‘Staying Alive’, to match the sad mentality behind a survival experiment. We also have a rule that it's okay to talk to the fish until they start responding. Then it's a compulsory coffee break time.

## References

[DMM052321C1] Uusi-Mäkelä, M., Harjula, S.-K. E., Junno, M., Sillanpää, A., Nätkin, R., Niskanen, M. T., Saralahti, A. K., Nykter, M. and Rämet, M. (2025). The inflammasome adaptor *pycard* is essential for immunity against *Mycobacterium marinum* infection in adult zebrafish. *Dis. Model. Mech.* 18, dmm052061. 10.1242/dmm.052061 39916610 PMC11972081

